# Microparticle-tagged image-based cell counting (ImmunoSpin) for CD4 + T cells

**DOI:** 10.1007/s00604-021-05070-y

**Published:** 2021-11-25

**Authors:** Sang-Hyun Hwang, John Jeongseok Yang, Yoon-Hee Oh, Dae-Hyun Ko, Heungsup Sung, Young-Uk Cho, Seongsoo Jang, Chan-Jeoung Park, Heung-Bum Oh

**Affiliations:** 1 Department of Laboratory Medicine, Asan Medical Center, University of Ulsan College of Medicine, Seoul, 05505 Republic of Korea; 2 Asan Institute for Life Sciences, Asan Medical Center, University of Ulsan College of Medicine, Seoul, 05505 Republic of Korea

**Keywords:** Human immunodeficiency virus, ImmunoSpin, Image-based cell counting, CD4 + T cell, Microparticle

## Abstract

Affordable point-of-care (POC) CD4 + T lymphocyte counting techniques have been developed as alternatives to flow cytometry-based instruments caring for patients with human immunodeficiency virus (HIV)-1. However, POC CD4 enumeration technologies can be inaccurate. Here, we developed a microparticle-based visual detector of CD4 + T lymphocytes (ImmunoSpin) using microparticles conjugated with anti-CD4 antibodies, independent of microfluidic or fluorescence detection systems. Visual enumeration of CD4 + T cells under conventional light microscope was accurate compared to flow cytometry. Microparticle-tagged CD4 + T cells were well-recognized under a light microscope. ImmunoSpin showed very good precision (coefficients of variation of ImmunoSpin were ≤ 10%) and high correlation with clinical-grade flow cytometry for the enumeration of CD4 + T cells (*y* = 0.4232 + 0.9485 × for the %CD4 + T cell count, *R*^2^ = 0.99). At thresholds of 200 and 350 cells/µL, there was no misclassification of the ImmunoSpin system compared to the reference flow cytometry. ImmunoSpin showed clear differential classification of CD4 + T lymphocytes from granulocytes and monocytes. Because non-fluorescence microparticle-tags and cytospin slides are used in ImmunoSpin, they can be applied to an automatic digital image analyzer. Slide preparation allows long-term storage, no analysis time limitations, and image transfer in remote areas.

**Graphical abstract:**

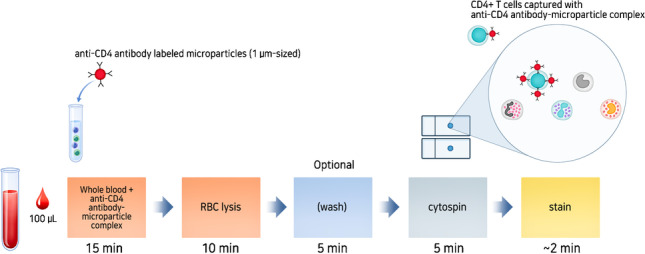

**Supplementary Information:**

The online version contains supplementary material available at 10.1007/s00604-021-05070-y.

## Introduction

The enumeration of CD4 + T cells in whole blood (WB) is an important test before initiating antiretroviral treatment and monitoring of treatment response [1] and disease progression [2]. The current reference technique for the enumeration of CD4 + T cells is flow cytometry which utilize fluorescence measurements of labeled antibodies to cell antigen markers at multiple wavelengths and light scatter [3]. It is a well-established method used to monitor immunomodulatory therapy and various applications in biomedical research, as well as CD4 + T cell monitoring in patients with HIV [4, 5]. Most flow cytometers are highly complex and expensive instruments that also require well-trained personnel. Thus, current technical challenges in the enumeration of CD4 + T cell counting for patients in resource-limited settings include simplicity, cost, environmental/infrastructure availability, technical requirement, accuracy, and precision [2, 6]. Rapid, reliable, and affordable point-of-care (POC) techniques including centrifugation, electrical, or fluorescence-based for CD4 + T cell counting have been developed [3].

The technical challenges of most fluorescence-based POC methods are related to the cost and the complexities of optical sensing components of filters, light sources, and detectors [7–9]. Despite the continued requirements of skilled operators and maintenance [10], the overall performance was generally lower than reference flow cytometry [11]. Moreover, to provide specific cell count of CD4 + T cells, two or more antibodies and fluorescent dyes had to be used to capture CD4 + CD3 + T lymphocytes. The electrical methods were compromised with imprecision and low signal-to-noise levels [12].

Several bright field (fluorescence-free) image-based techniques were simple approaches but showed relatively underperformance that potentially harbored false positive results due to CD4 + monocyte [13]. These microfluidic approaches depend on the efficiency and specificity of CD4 + T cell isolation and capture as discrimination from other CD4 + cells such as monocytes are important. Another important problem was the inability to report the percentage of CD4 + T cells (CD4 + T cells / total lymphocytes × 100) by most CD4 + T cell counters using capture and isolation principle. The variation of CD4 + percentage is smaller than the absolute number of CD4 + T cells, and thus, the measurement of %CD4 is important especially in pediatric patients. Otherwise, to report both CD4 + T cell percentages and absolute CD4 + T cell count would require simultaneous detection of two or more types of cells using multiple fluorescent antibodies [8].

To overcome the complex optics requirement in fluorescence imaging and improve the analytical performance of counting CD4 + T cells as well as CD4 + percentage, we focused on the following technical challenges: (1) no capture and isolation steps without microfluidic instrumentation, (2) fluorescence-free detection, (3) accurate results of both absolute CD4 + T cell number and percentage using only single anti-CD4 antibody, and (4) using only commercially available materials and components for the method.

We conceptualized that the use of microparticles to label specific cell antigens as alternatives to fluorescent dyes and preparation on glass slides can accomplish image-based specific cell counting (CD4 + T cells in this study) under conventional light microscope or digital morphology hematology analyzers that incorporate blood smear slides. Among several choices of microparticles as an alternative to fluorescent dye, commercial magnetic particles used in this study fit the purpose of excellent visual recognition under light microscope. Commercially available microparticles have significant advantages in minimizing bead-to-bead variation associated with functionalization, reproducibility of results, and material affordability.

Described here is a newly developed microfluidics‐free/fluorescent dye-free concept test (microparticle-tagged image-based cell counting, shortly, ImmunoSpin) for the CD4 + T cell counting. Using microparticles labeled with anti-CD4 antibody alone, test result can be identified under a conventional light microscope without signal generation. ImmunoSpin utilizes cytocentrifugation (not to isolate the cells) and provides both the %CD4 + T cells and the absolute number of CD4 T cells. Moreover, slide preparation of magnetic bead-tagged images provides long-term storage, removal of analytical time limit, and transferrable images to other experts in remote areas. Digital morphological analysis of blood smears has been a developing field due to recent advances in digital imaging and information technology [14]. Microparticle-tagged image-based CD4 + T cell counter is a promising concept that has the potential to be applied in digital image analyzer in the future.

## Materials and methods

### Functionalization of the beads with antibodies: anti-CD4 antibody–particle complex solution

Streptavidin-coated microparticles (10 mg/mL, mean diameter 1 μm, Dynabeads MyOne Streptavidin C1, Life Technologies, Grand Island, NY, USA) were conjugated with biotinylated anti-CD4 antibody (RPA-T4, eBioscience, San Diego, CA, USA). Briefly, 5 µL of 1:10 diluted streptavidin-coated microparticles was gently mixed with 3 μL of 1:10 diluted monoclonal anti-CD4 antibody (0.2 mg/mL, Invitrogen) for 10 min before use. Titration of anti-CD4 antibodies and microparticles was performed (Figs. S1 and S2).

### ImmunoSpin: CD4 + T cell labeling with the anti-CD4 antibody–microparticle complex

WB samples were collected from patients at the Asan Medical Center from May 2020 to April 2021. After a routine lymphocyte subset test with flow cytometry or routine human leukocyte antigen cross-match tests, the leftover or residual WB or peripheral blood mononuclear cell (PBMC) samples were used. PBMC samples were used temporarily at the start of optimization, especially for rapid identification without fixing and staining. The experiment was approved by the Ethical Review Board of the Asan Medical Center (IRB No. 2020–0222).

**Fig. 1:**
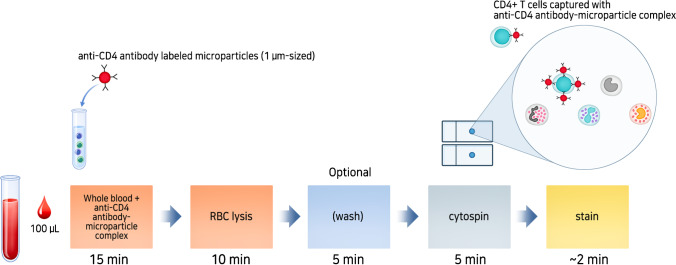
Process illustration for microparticle-tagged image-based cell counting (ImmunoSpin). Representation of specific labeling of CD4 + T cells from whole blood (WB) with the anti-CD4 antibody–microparticle complex and their subsequent RBC lysis and washing step (optional). Then, cytospin preparations were established for the enrichment of leukocytes, including anti-CD4 antibody–microparticle-labeled lymphocytes. Cytospin slides were counterstained with Wright (or methylene blue) stain. At least two cytospin slides were prepared from 100 µL WB and were analyzed

After optimization, CD4 T cell levels were measured in unfractionated WB from patients. WB was incubated with anti-CD4 antibody–microparticle complexes (Fig. 1). Briefly, 100 μL of WB was added to a tube and captured with an anti-CD4 antibody–microparticle complex and incubated at room temperature for 15 min. Then, erythrocytes (RBCs) were lysed with 500 μL of BD lysing buffer (BD Biosciences, San Jose, CA, USA) for 10 min. After removing the supernatant, the cell pellet was suspended in 10% polyethylene glycol 8000 (PEG8000, Sigma-Aldrich, Saint Louis, MO, USA) BD FACS buffer (final 800 µL), which was ready for cytospin.

According to routine preparation of cytospin films, a suspension of cells (100 µL) tagged with the anti-CD4 antibody–microparticle complex was loaded onto cytospin cuvettes for cytospin [15] and counterstained. The detailed preparation of cytospin films was shown in the Electronic Supporting Material (ESM).

### Enumeration of cytospin films of ImmunoSpin

ImmunoSpin did not alter the proportion of leukocyte differentials, including granulocytes, monocytes, and lymphocytes, due to the lack of selective isolation of specific cells during processing (Supplementary Table S1). Thus, the proportions of CD4 + T cells can be directly calculated by counting the total lymphocytes and microparticle-labeled CD4 + T cells. As the absolute count of CD4 + T cells is easily derived from the dual-platform method [16], we focused on the accuracy of the proportion of the CD4 + T cell count by ImmunoSpin. The detailed calculation of absolute count of CD4 + T cells was shown in the ESM. %CD4 + T lymphocyte enumeration of ImmunoSpin was carried out by two skilled hematologists on 400 cells at 400 × magnification.

### Effect of additives (PEG8000 and Ficoll) and FACS buffer on ImmunoSpin

Whether additives, such as Ficoll70 (Ficoll70, Sigma-Aldrich, St. Louis, MO, USA) and PEG8000, improved the preservation of cell morphology and the stability of cells on the ImmunoSpin system in addition to FACS buffer (BD) was evaluated. Optimization conditions with PEG8000 at several concentrations (5%, 10%, and 25%) were evaluated for understanding the morphology of cells and the binding aspect of the antibody–microparticles complex with target cells (Figs. S3 and S4). Based on the results of Wong et al. [17], 10% Ficoll70 was tested in this study.

### Reference method: flow cytometric detection of CD4 + T cells

The proportion of CD4 + T cell (%CD4 + T cells) on FACSCanto II flow cytometry (BD Biosciences, San Jose, CA, USA) by analyzing 30,000 events by the lymphocyte gate was measured as a reference method using the BD Multitest six-color TBNK reagent (CD3 FITC/CD16 PE + CD56 PE/CD45 PerCP-Cy5.5/CD4 PE-Cy7/CD19 APC/CD8 APC-Cy7) [18]. The reference flow cytometry instrument was calibrated daily and checked by running Multicheck Normal and Low controls (BD Biosciences). The results were analyzed using FACS Diva software (BD Biosciences) for routine CD4 + T cell counting in a clinical laboratory.

### Precision and limit of detection

The coefficient of variation (CV) was calculated as the standard deviation divided by the means of repeated measurements and was used for precision. The within-run and between-run imprecision of ImmunoSpin were assessed on duplicate measurements of each run and two runs per day over 5 consecutive working days (total 20 replicates) of Multicheck Normal (BD Biosciences, Lot BM1120N, lymphocyte count 1488 cells/µL) and low controls (BD Biosciences, Lot BM11202L, lymphocyte count 1,103 cells/µL), respectively. Within-run imprecision was performed using 12 samples, displaying different leukocyte counts. The limit of detection (LOD) was calculated as the limit of blank (LOB) + 1.654 * standard deviation (SD) (shown in the ESM) [19].

### Linearity and bias of ImmunoSpin

Linearity or spiked recovery was assessed by spiking the Multicheck Normal controls (BD Biosciences) with very low (0.2% lymphocyte) CD4 patient samples in six different ratios (5:0, 4:1, 3:2, 2:3, 1:4, and 0:5), and the results were obtained by establishing the corresponding CD4 counting results using a BD FACSCanto II flow cytometer (BD Biosciences, San Jose, CA, USA) as the reference method. Analyses were performed in duplicates. The agreement between ImmunoSpin system results and those obtained by flow cytometry was assessed by Passing–Bablok regression analysis. Bias was evaluated by comparing CD4 + T cell count results of the ImmunoSpin to those of the reference BD FACSCanto II flow cytometer (BD Biosciences) using the Multicheck Normal and Low process controls (BD Biosciences).

### Comparison and misclassification at 200 and 350 cells/μL thresholds of clinical importance

Total WB samples (*n* = 45) were used for comparison. Absolute CD4 + T cell counts and CD4 T cell proportions measured by ImmunoSpin were compared to those of the BD FACSCanto II flow cytometer (BD Biosciences). The absolute count of CD4 + T cells was calculated for the evaluation of the method of comparison and misclassification [16]. The results were then analyzed using Passing–Bablok regression and Bland–Altman plot analysis. Misclassification of CD4 + T cell counts was performed at clinically relevant CD4 + T cell thresholds of 200 and 350 cells/μL, using flow cytometry as the “true” value [20]. Statistical analyses were performed using MedCalc version 18.2.1 (MedCalc Software, Mariakerke, Belgium).

## Results and discussion

### ImmunoSpin

We developed a novel fluorescence-free simple visual detection system (ImmunoSpin) that incorporates specific antibody-labeled microparticles (Dynabeads MyOne, Life Technologies), leading to the accurate detection of CD4 + T cells under a conventional light microscope. This microparticle-tagged image-based CD4 count will be very useful in resource-limited settings because it can be performed without additional sophisticated instrumentation related to signal generation, calibration, and optical detection. Figure 1 shows a schematic of the microparticle-tagged image-based cell counting approach (ImmunoSpin). After RBC lysis, ImmunoSpin could also perform the cytospin step without washing. When ImmunoSpin was performed without the RBC wash step, microparticle-tagged CD4 + T cells were well-recognized under a light microscope (Fig. S5).

Dynabeads MyOne (Life Technologies) is a magnetic microparticles (1 μm; size distribution, CV < 3%) with streptavidin monolayer covalently coupled to the hydrophilic bead surface, which has wide range of applications for biological assays [21, 22]. Dynabeads MyOne is made of porous polymeric spheres evenly embedded with iron oxide [23]. These magnetic microparticles were shown to be compatible with visual identification without an optical instrument for the detection of DNA through our previous works [24, 25].

We utilized the cytocentrifuge for the enrichment of WBCs, which is adapted in most pathology laboratories for cytologic analysis, even in resource-limited settings. The cytospin technique involves thin-layer preparation through a cytocentrifugation process from liquid materials, especially those containing low cell numbers, such as cerebrospinal fluid or pleural effusions [26]. We adapted this cytospin technique for cell concentration instead of CD4 + T cell isolation and concentration techniques. The utilization of well-established cytocentrifugal cell preparation is an important advantage of ImmunoSpin. Certain flow cytometry or fluorescence-free new-generation cell counting technologies require additional strategies for the isolation or enrichment of target cells. Magnetic capture [27], antibody-based capture [6], or microfluidic pillar structures [28] were inevitably used to isolate or enrich specific target cells, including CD4 + T cells. Thus, the capture efficiency and accuracy of CD4 + T cell counting were greatly affected by the microfluidic chip design and flow rate [6]. Importantly, most CD4 + T cell counters using capture and isolation of CD4 + T cells are unable to report the percentage of CD4 + T cells. One of promising POC CD4 + T cell counting strategy is a smartphone based cytometric techniques which can be inexpensive, simple, and rapid [29]. However, using only CD4-antibody and the lack of procedures to differentiate CD4 + monocytes can be a complication. Visual confirmation and long-term storage are also not possible. Most smartphone cytometry platforms are still limited by the throughput [30]. We have summarized the specific features of ImmunoSpin compared with several portable cytometries in supplementary Table S3.

To our knowledge, ImmunoSpin is the first microparticle-based image CD4 + T cell counter that uses only one antibody and the non-flow/non-fluorescence image method. To ensure specificity and calculate the %CD4 + T cells, we utilize the cell morphology (WBC differential) and microparticle-labeled characteristics of cells. One of the main advantages of ImmunoSpin is that there is no isolation of targeted cells (the magnetic particles were just used as labels.). Therefore, we can calculate the proportion of CD4 + T cells (%CD4 + T cells) and the absolute number of CD4 + T cells using *only one antibody* (most CD4 + T cell counters with a cell isolation step cannot report the %CD4 + T cells). %CD4 + is the preferred method of diagnosis for patients under 5 years of age. ImmunoSpin is prepared as cytofilm and permanently stored at room temperature. Therefore, the remote review of ImmunoSpin from another location allows for the implementation of tele-hematology in routine hematological laboratories.

### Clear differentiation of CD4 + T cells from monocytes

**Fig. 2:**
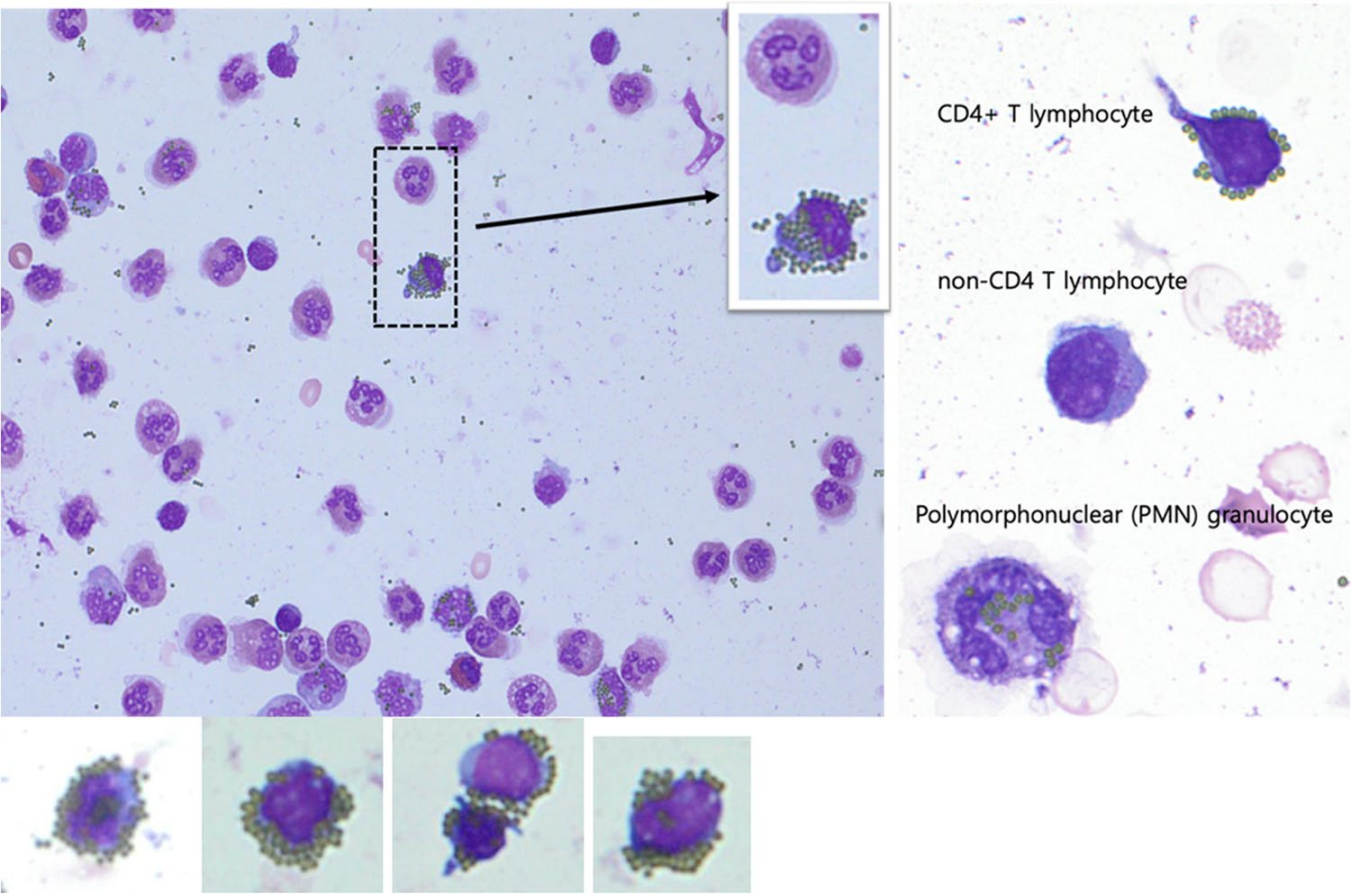
Microscopy images indicating the CD4 + T cells by anti-CD4 antibody-conjugated microparticles and a granulocyte (dashed box) in the background of other leukocytes (above). Several morphologies of microparticle-tagged CD4 + T cells are illustrated (below)

In ImmunoSpin, CD4 + T cells were easily differentiated morphologically from monocytes which express CD4 antigens and cause falsely increased CD4 + T cell enumeration (Fig. 2). Thus, monocytes were excluded for CD4 + T cell enumeration in ImmunoSpin (Fig. S6). The position and patterns of microparticle-tagged lymphocytes were differentiated from those of monocytes and other non-specific microparticle-binding granulocytes. For CD4 + T cells, microparticles were tagged along the cell membrane surface. However, microparticles were observed in the cytoplasmic area of monocytes and other non-specific binding granulocytes. For certain instruments, such as micro-a-fluidic ELISA [31], the flow-through cell counting assay [32], which involves colorimetric detection, can yield false positives due to the interference of monocytes on the CD4 + T cell count [33]. Some instruments require additional strategies, such as anti-CD3 antibodies, for detection to avoid signals from captured monocytes [6]. Additionally, this ImmunoSpin technology can be easily implemented for automatic (artificial intelligence) image analysis with slide scanning or integrated for a fully automated microfluidic CD4 analyzer and manual counting under a light microscope.

### Effect of PEG8000 and ficoll70 on cell morphology

We evaluated whether additives improve the preservation of the cell morphology of leukocytes after the removal of debris and RBC lysis buffer through which WBC was subsequently concentrated by cytocentrifugation. After optimization with PEG8000 at several concentrations (5%, 10%, and 25%) (Fig. S3), neutrophil morphology was better preserved in a 10% PEG8000-stabilized cell suspension than in a 10% Ficoll70-stabilized cell suspension (Fig. S7). PEG8000 and FACS buffer substantially decreased the degradation of the cell suspension at 24 h, and the cell morphology and cells tagged with antibody–microparticle complexes were more clearly identifiable (Figs. S8 and S9).

PEG decreases lipid peroxidation and swelling and, to protect the cell cytoskeleton from cold [34], reduces cell apoptosis by protecting cell membranes and mitochondria and inhibiting free radicals [35]. Thus, PEG stabilizes the lipids in the membrane. The effects of PEG appear to be related to the preservation of cell shape [36, 37]. In this study, 10% PEG8000 was effective for the preservation of cell morphology. Although Ficoll 70 kDa (Ficoll70) stabilized blood samples [17], we did not observe any additional effect on cell morphology. Because we did not focus on cell preservation before analysis, we did not extensively evaluate the effect of Ficoll70.

CD4 + T cell counts were stable up to at least 36 h after the test mixture preparation of anti-CD4 antibody–microparticles and cells, even at room temperature with 10% PEG8000. Moreover, our ImmunoSpin preparation as cytospin slides was excellent for long-term storage because the cytocentrifuged cytospin slides were fixed and permanently mounted. This is advantageous for remote transport or re-analysis with other automated scanning or expert reviews. Long-term storage or re-analysis is not possible using the flow cytometric approach.

### Precision and limit of detection

The ImmunoSpin method showed excellent analytical performance. The repeatability and between-run CV of ImmunoSpin using Multicheck Low CD4 control (BD Bioscience, Lot BM11202L) were 4.1% and 7.1%, respectively, and the repeatability and between-run CV of ImmunoSpin using Multicheck Normal CD4 control (BD Bioscience, Lot BM1120N) were 1.0% and 2.1%, respectively (Table 1). All CVs of ImmunoSpin were ≤ 10% for low counts (< 20% of CD4 T cell control materials) and ≤ 5% for high counts (> 40% of CD4 T cell control materials). A CV below 5% was considered optimal, and that between 5 and 10% was considered acceptable [38]. The WHO Prequalification of Diagnostics Programme (PQDx) accepts a maximum relative bias of 10% (e.g., 500 ± 50 CD4 T cells) for a CD4 T cell count [2]. The LOB and LOD of ImmunoSpin was 0.4% and 2.6%, respectively (the details were shown in ESM).

**Table 1:** Precision of the ImmunoSpin system

	Mean %CD4 T cells of multicheck controls by ImmunoSpin (20 replicates)	Repeatability (%CV)	Between-run (%CV)	Within laboratory (between days, %CV)
Multicheck Low CD4 control	12.2% (135 cells/μL)	4.1%	7.1%	8.2%
Multicheck Normal CD4 control	48.0% (714 cells/μL)	1.0%	2.1%	2.4%

### Linearity and bias of the ImmunoSpin system

The linearity of ImmunoSpin was very good, with *R*^2^ = 0.986 (*y* = 0.9056*x* + 1.9375). The assessments of spiked recovery were shown in supplementary Table S2. The mean bias between ImmunoSpin and flow cytometry is shown in Table 2. The mean bias was − 0.6% for the low CD4 control material and − 0.5% for the normal CD4 control material.

**Table 2:** The bias between ImmunoSpin and flow cytometry

%CD4 T cell	Mean%CD4 T cells by ImmunoSpin	%CD4 T cells by FACSCanto II	Mean bias	Relative bias
Multicheck Low CD4 control material (Lot. BM11202L) (3 replicates)	**12.2% ± 0.8 (135 cells/μL)**	**12.8% ± 0.1 (141 cells/μL)**	− 0.6%	− 4.6%
Multicheck Normal CD4 control material (Lot. BM1120N) (4 replicates)	**48.0% ± 1.1 (714 cells/μL)**	**48.5% ± 0.6 (722 cells/μL)**	− 0.5%	− 1.0%

### Comparisons

**Fig. 3:**
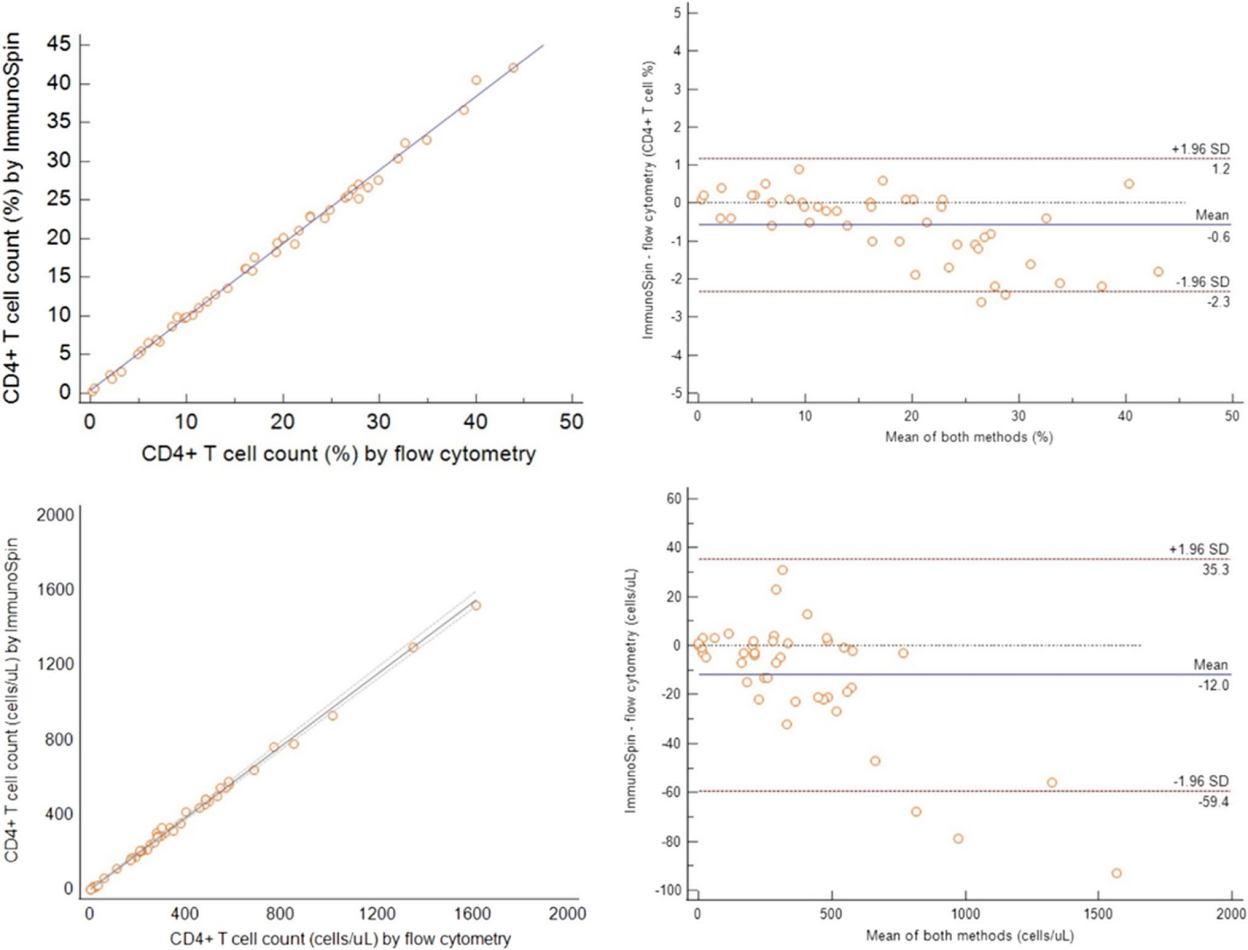
Comparison of ImmunoSpin with flow cytometry. Total WB samples (*n* = 45) were used for method comparison. CD4 + T cell proportions (above) and absolute CD4 T cell counts (below) measured on the ImmunoSpin were compared to the corresponding CD4 + T cell counting from the flow cytometer BD FACSCanto II (BD Biosciences). The regression equation was *y* = 0.4232 + 0.9485 × for %CD4 + T cell count (*R*^2^ = 0.99) and *y* = 4.1001 + 0.9590 × for absolute CD4 + T cell count (*R*^2^ = 0.99). The slopes were 0.95 (95%CI, 0.93–0.97) for %CD4 + T cell count and 0.96 (95%CI, 0.94–0.99) for absolute CD4 + T cell count. Absolute differences calculated with Bland–Altman plot analysis are plotted

In this study (*n* = 45), 11 WB from patients with < 200 CD4 + T cells/μL, 23 from patients with 200–500 CD4 + T cells/μL, and 11 from patients with > 500 CD4 + T cells/μL were compared (Fig. 3). The regression equation was *y* = 0.4232 + 0.9485 × for the %CD4 + T cell count (*R*^2^ = 0.99) and *y* = 4.1001 + 0.9590 × for the absolute CD4 + T cell count (*R*^2^ = 0.99). The slopes were 0.95 (95% CI, 0.93–0.97) for % CD4 + T cell count and 0.96 (95% CI, 0.94–0.99) for absolute CD4 + T cell count.

As with ImmunoSpin, available manual alternatives for flow cytometry for CD4 + T cell enumeration in resource-limited laboratories are VISITECT CD4 and Dynal T4 Quant (Dynal Biotech Oslo, Norway) [8, 39]. VISITECT CD4 is instrument-free but not quantitative (semi-quantitative test based on lateral-flow technology). The Dynal T4 Quant kit uses magnetic Dynabeads to isolate specific cells and needs a fluorescent microscope although it can be performed with a light microscope [39, 40]. In addition, it cannot identify lymphocytes or the CD4 + T cell % of lymphocytes [41]. At the level of CD4 (200–499 cells/µL), the correlation of Dynal T4 Quant was relatively low compared to that of flow cytometry (*r* = 0.797)[41]. Once lysed, cells must be analyzed within 1 h [41].

### Clinical misclassification

At the thresholds of 200 and 350 cells/µL, there was no misclassification of the ImmunoSpin system compared to flow cytometry (Table 3). The sensitivity and specificity of the ImmunoSpin were 100% (71.5–100%), and the specificity was 100% (89.7–100%), respectively, with a kappa coefficient of 1.0. The performance of the ImmunoSpin was comparable to that of the PIMA CD4 (Alere Technologies) of which the sensitivity and specificity were 92% (95% CI, 88–95%) and 87% (95% CI, 85–88%), respectively [11]. The WHO prequalified PIMA CD4 (Alere Technologies) is a fluorescent image cytometer that requires surface antibody immobilization and fluorescent labeling [2].

**Table 3:** Clinical misclassification at 200 and 350 cells/μL thresholds of clinical importance

	Threshold at 200 cells/μL (flow cytometry)		Threshold at 350 cells/μL (flow cytometry)	
ImmunoSpin	< 200 cells/μL	> 200 cells/μL	< 350 cells/μL	> 350 cells/μL
< threshold	11	0	27	0
≥ threshold	0	34	0	18

## Conclusion

In this study, we demonstrated the feasibility of a simple, fluorescence-free/image-based cell counting assay for the enumeration of CD4 + cells using antibody-labeled microparticles, which were detected by light microscopy or image analysis. We demonstrated good analytical performance of the ImmunoSpin and high correlation with clinical-grade flow cytometry for the enumeration of CD4 + T cells. ImmunoSpin is more affordable and easier to perform than flow cytometry or new-generation fluorescence-based microfluidic instruments. Centralized lab-based flow cytometers are sophisticated, have high operational and maintenance costs, and require skilled technicians [8, 32].

POC CD4 + T cell instrument options are currently lacking. This is partly due to technological challenges, including the need for efficient CD4 isolation, exclusion of cross-contamination in signals from monocytes (which also express CD4 surface marker), and delicate microfluidic control [6]. The ImmunoSpin system overcomes the above challenges as follows: efficient CD4 + T cell concentration by simple cytocentrifugation, no cross-contamination from monocytes due to image-based analysis, and no need for sophisticated flow control. In addition, ImmunoSpin does not require a wash step to remove RBC lysis and other debris because the image-based analysis of ImmunoSpin can identify cells in the presence of lysed RBCs and debris.

Limitations of ImmunoSpin at this stage include the following: (1) requires manual processing [42]. As with other technologies, lysing or removing RBCs reduces cell counting errors due to the higher concentration of RBCs [3]. ImmunoSpin needs cytocentrifugation and microscope; (2) at the very beginning of the developmental stage, multiplexing detection of various cell surface antigens in one tube, such as immunophenotyping, is required, which utilizes various fluorescence dyes at the same time as flow cytometry[43]; and (3) the optimal size of the microparticles for ImmunoSpin on a light microscope was 1–2 µm. The use of larger-sized microparticles (4.5 µm in diameter) resulted in aggregation with CD4 + T cells (Fig. S10).

ImmunoSpin could help resource-limited laboratories to quantitatively determine CD4 + cell counts without sophisticated instrumentation. Cytospin preparation for ImmunoSpin can be applied through staining and long-term (permanent) storage and remote transportation for review or re-analysis. Further development of the assay will provide great potential not only for CD4 + T cell enumeration but also for the quantification of other immune cells, such as CD8 + T cells, B cells, and NK cells, as long as target-specific surface marker antibodies are used in resource-limited settings.

In the near future, for true POC use, we intend to develop a new version of ImmunoSpin integrated with microfluidics for tagging CD4 + T cells with the anti-CD4 antibody–microparticle complex on the chip, as well as for the washing and delivery of antibody-labeled microparticles, with an integrated AI-based image analysis.

## Supplementary Information

Below is the link to the electronic supplementary material.

Supplementary file1 (DOCX 40863 KB)
