# Supplementary material for: Microparticle-tagged image-based cell counting (ImmunoSpin) for CD4 + T cells
Source: Mikrochim Acta. 2021 Nov 25;188(12):431. doi: 10.1007/s00604-021-05070-y (PMC8616869; doi:10.1007/s00604-021-05070-y)
Supplement: Supplementary file 1 — Supplementary file1 (DOCX 40863 KB) [file 604_2021_5070_MOESM1_ESM.docx]

**Electronic Supplementary Material**

**Microparticle-tagged image-based cell counting (ImmunoSpin) for CD4+ T cells**

Sang-Hyun Hwang^1,2^, John Jeongseok Yang^1^, Yoon-Hee Oh^1^, Dae-Hyun Ko^1^, Heungsup Sung^1^, Young-Uk Cho^1^, Seongsoo Jang^1^, Chan-Jeoung Park^1^, Heung-Bum Oh^1*^

^­^

^1^Department of Laboratory Medicine, Asan Medical Center, University of Ulsan College of Medicine, Seoul, 05505, Republic of Korea

^2^Asan Institute for Life Sciences, Asan Medical Center, University of Ulsan College of Medicine, Seoul, 05505, Republic of Korea.

^*^ To whom correspondence should be addressed;

Tel: +82-2-3010-4505; Fax: +82-2-478-0884; E-mail address: [hboh@amc.seoul.kr](mailto:hboh@amc.seoul.kr)

**Cytospin preparations for the enrichment of leukocytes, including anti-CD4 microparticle-labeled T cells**

A suspension of cells (100 µL) tagged with the anti-CD4 antibody–microparticle complex was loaded onto cytospin cuvettes, mounted onto glass slides and paper cards, and centrifuged at 800 × *g* for 5 min to deposit a monolayer of cells on the slides [[1](#_ENREF_1)]. Slides were counterstained with Wright stain (YD Wright Stain, YD Diagnostics, Yongin, Korea) according to the manufacturer’s instructions, and the cell types and differential counts were identified under a light microscope. At least two cytospin slides were prepared from 100 µL of WB (Figure 2). If necessary, especially for rapid checks, methylene blue stain (Sigma-Aldrich, St. Louis, MO, USA) was also performed during optimization.

**Enumeration of cytospin films of ImmunoSpin**

Briefly, for absolute lymphocyte counts per microliter of WB by the dual‐platform method, the proportion of CD4+ T cells derived by flow cytometry was multiplied by the absolute lymphocyte count derived from the automated hematology analyzer [[2](#_ENREF_2)].

**Limit of blank (LoB) and Limit of Detection (LoD)**

LoB was determined by two replicates of 5 samples for a total of 10 measurements. LoD was determined as LoB + 1.645 * standard deviation(SD)_blank samples_ [[3](#_ENREF_3)]_._ Ten replicates of three low concentration samples with results of 4.4 %, 6.6 % and 9.9 % were measured (a total of 30 measurements) for SD_low positive samples_.

Supplementary Table S1. Comparison of white blood cell distribution between CBC analyzer and ImmunoSpin (n = 10)

|  | CBC analyzer | ImmunoSpin | *P* value* |
| --- | --- | --- | --- |
| Granulocyte | 54 ± 15 | 51 ± 14 | 0.138 |
| Lymphocyte | 36 ± 13 | 37 ± 13 | 0.138 |
| Monocyte | 7 ± 1 | 8 ± 1 | 0.217 |
| Eosinophil | 3 ± 2 | 3 ± 2 | 0.752 |
| Basophil | 1 ± 0 | 1 ± 1 | 0.832 |

*paired-t test

**Supplementary Table S2. Determination of recovery from whole blood samples spiked with CD4+ T cell control materials**

| Spiked volume | Expected %CD4+ T cells | Measured %CD4+ T cells (± SD) | Recovery (%) |
| --- | --- | --- | --- |
| Multicheck Normal control 100uL | 49.0 | 45.4 ± 4.8 | 92.6 |
| Multicheck Normal control 80uL +  sample matrix 20uL | 39.2 | 39.3 ± 3.0 | 100.1 |
| Multicheck Normal control 60uL +  sample matrix 40uL | 29.4 | 26.4 ± 1.5 | 89.9 |
| Multicheck Normal control 40uL +  sample matrix 60uL | 19.6 | 20.3 ± 1.4 | 103.6 |
| Multicheck Normal control 20uL +  sample matrix 80uL | 9.8 | 13.4 ± 1.4 | 136.5 |
| sample matrix 100uL | 0.0 | 0.0 | NA |

NA; not applicable

**Supplementary Table S3. Specifications of POC CD4 test technologies**

|  | Signal / labeling | Technology | Sensitivity at 200 – 500 cells per μL | Specificity at 200 – 500 cells per μL | %CV | %CD4+ T cell | Detection time | LOD | Cost per test | Marketed |
| --- | --- | --- | --- | --- | --- | --- | --- | --- | --- | --- |
| ImmunoSpin | One microparticle-labelled antibody | Image-based | 100% | 100% | < 10 % | Yes | ~30 min | 2.6 % | < $1 | No |
| Smartphone-based CD4 testing [[4](#_ENREF_4)] | One antibody | Smartphone -based cytometry without flow | 100% | > 90% | NA | No | 30 min | 60 cells per μL | < $5 | No |
| Alere PIMA CD4 [[5-8](#_ENREF_5)] | Two fluorescent dye-labeled antibodies | Image-based cytometry | 92 % | 87% | < 15 % | No | 20 min | NA | $ 8.7 | Yes |
| CyFlow miniPOC cytometer [[9](#_ENREF_9), [10](#_ENREF_10)] | Two fluorescent dye-labeled antibodies | Flow cytometry | >95% | >94% | <7% | Yes | 17 min | NA | $ 3.96 | Yes |

**NA; not applicable**

| 5 µL of 1:10 diluted anti-CD4 antibody–microparticle complex | 1 µL of 1:10 diluted anti-CD4 antibody–microparticle complex | 1 µL of 1:100 diluted anti-CD4 antibody–microparticle complex |
| --- | --- | --- |
| 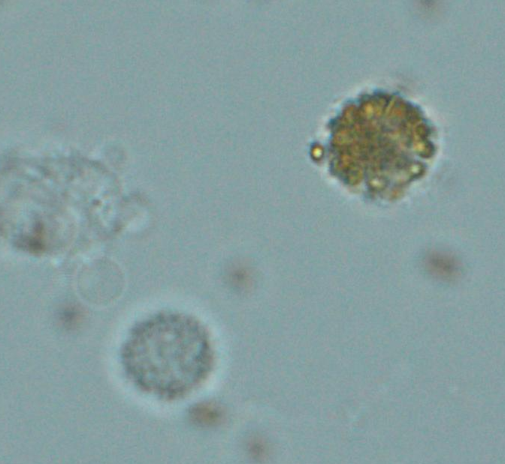Microparticle density on cells: +++ | 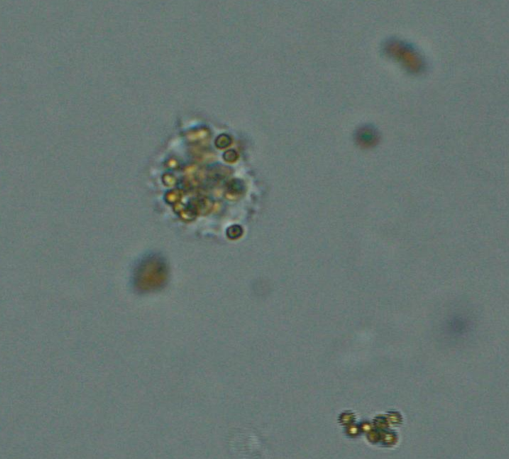  Microparticle density on cells: ++ | 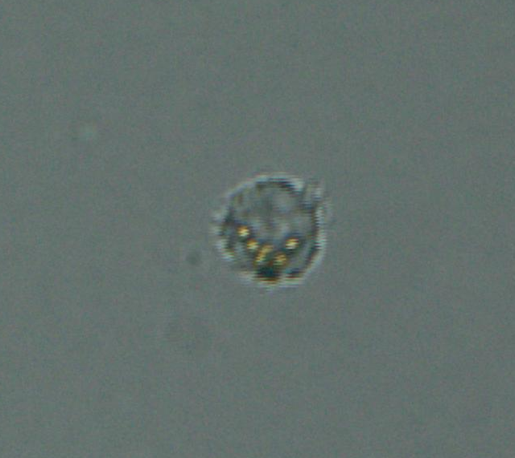  Microparticle density on cells:  + |

Supplementary Figure S1. Optimization of microparticle concentration. The maximum binding was reached for 5 µL (5 µg) of 1:10 diluted microparticles. Titration of the microparticles (10 mg/mL, mean diameter of 1 μm, Dynabeads MyOne Streptavidin C1, Life Technologies, Grand Island, NY, USA) was performed for concentrations of 0.1–5.0 µg, where the maximum binding was reached for 5 µL (5 µg) of 1:10 diluted microparticles. Peripheral blood mononuclear cells were purified from the whole blood using Ficoll separation. Slides without fixation and staining were prepared for analysis under a light microscope (1000x).

| 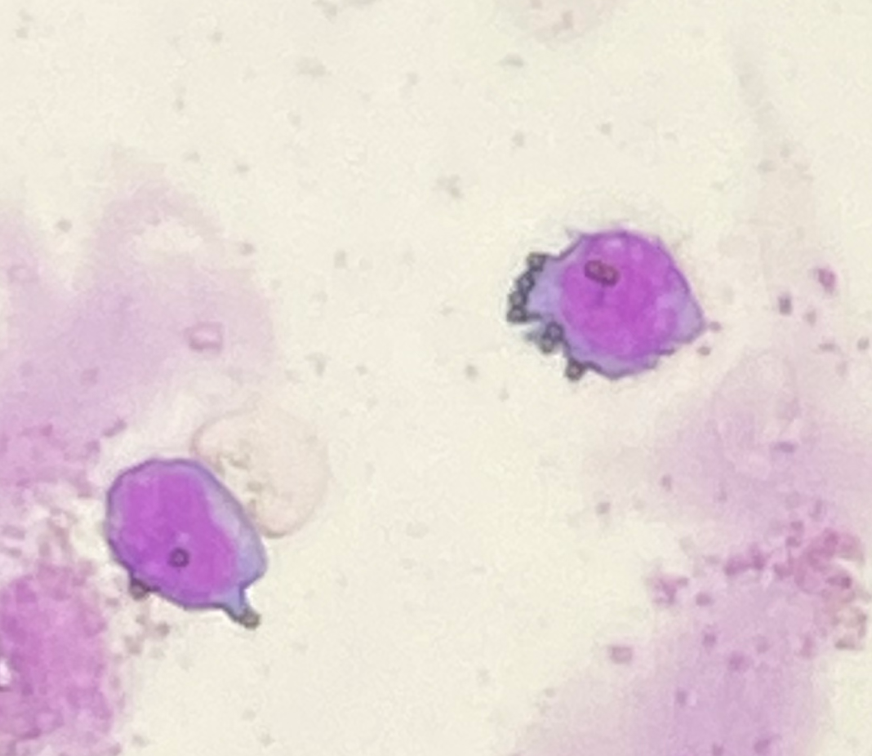 | 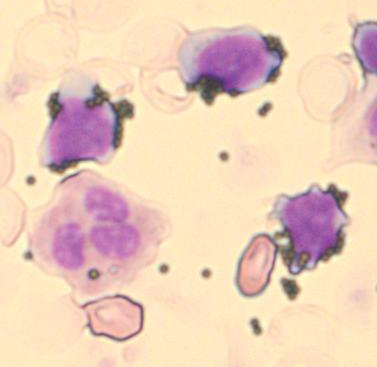 |
| --- | --- |
| 0.5 µL of 1:10 diluted anti-CD4 antibody | 1 µL of 1:10 diluted anti-CD4 antibody |
| 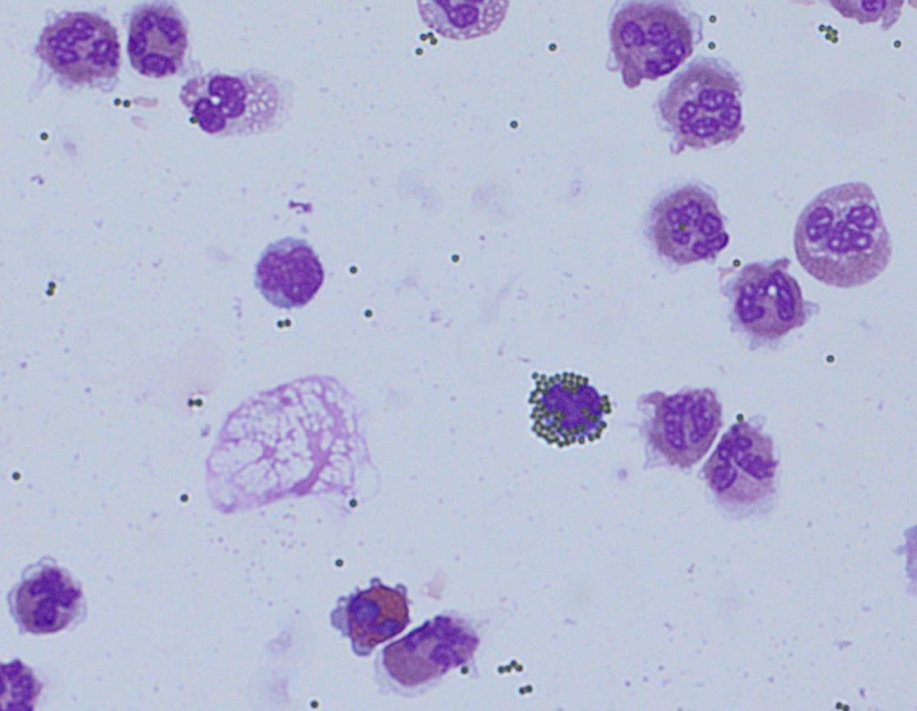 |  |
| 3 µL of 1:10 diluted anti-CD4 antibody |  |

Supplementary Figure S2. Optimization of anti-CD4 antibody titrations. The optimal reaction volume of anti-CD4 antibody was 3 µL. Titration of the anti-CD4 antibody (RPA-T4, eBioscience, San Diego, CA, USA) was performed for 0.5, 1, and 3 µL of 1:10 diluted anti-CD4 antibody. The optimal reaction volume of anti-CD4 antibody was 3 µL (the concentration of microparticles was fixed at 5 µL of 1:10 diluted microparticles). Slides were counterstained with Wright stain (YD Wright Stain, YD Diagnostics, Yongin, Korea).

| 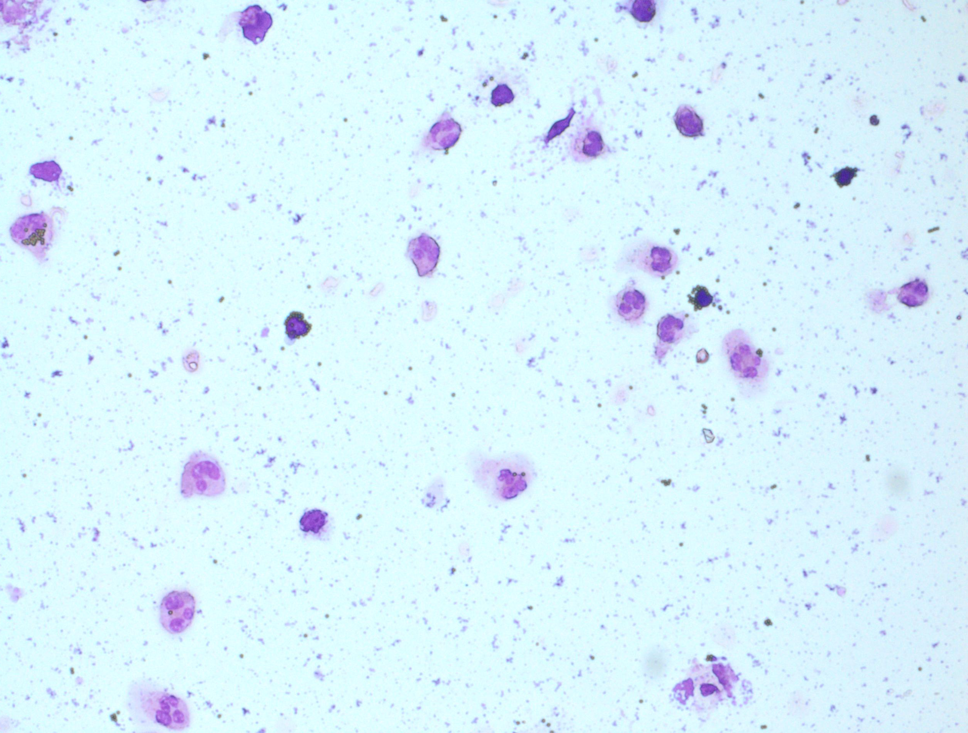 | 5% PEG8000 (Wright staining, 400x) |
| --- | --- |
| 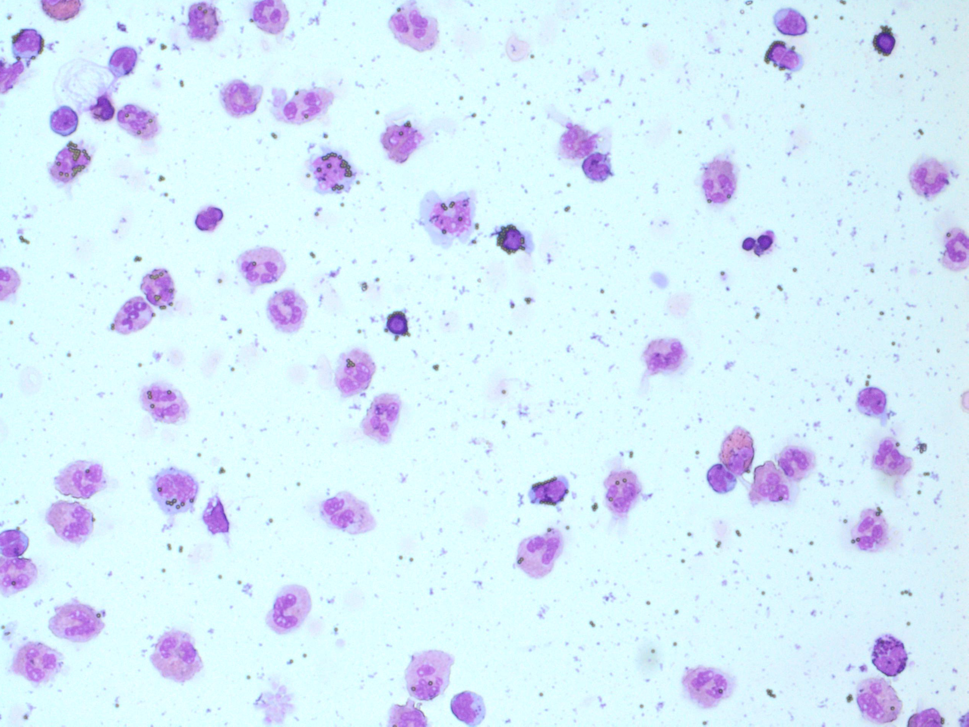 | 10% PEG8000 (Wright staining, 400x) |
| 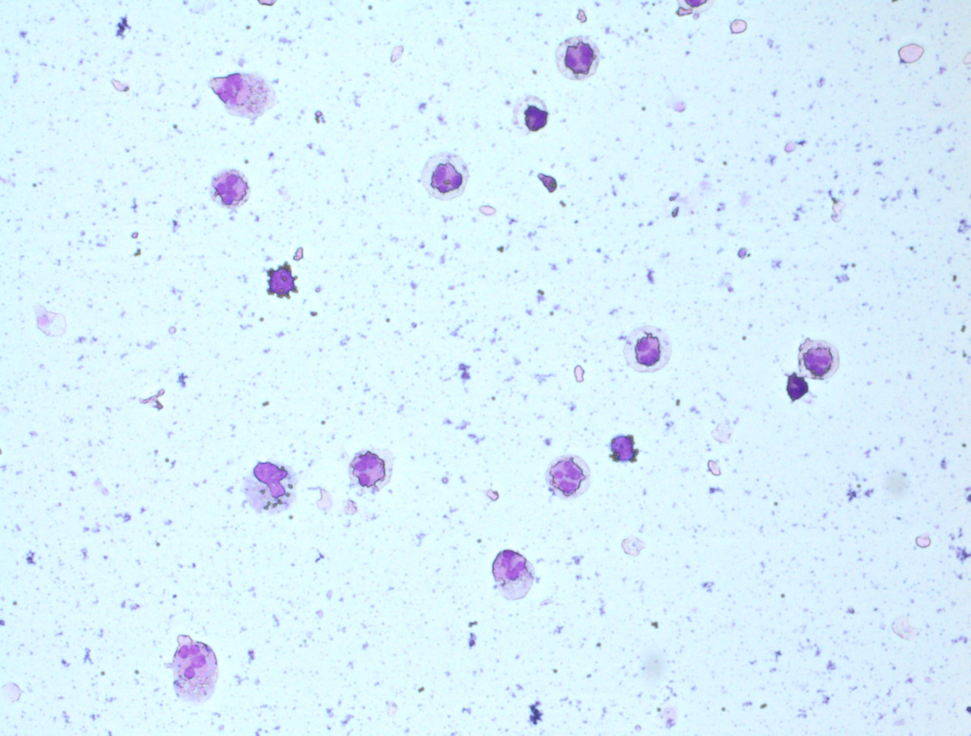 | 25% PEG8000 (Wright staining, 400x) |

Supplementary Figure S3. Determination of the optimal PEG8000 concentration. Before the cytospin step of ImmunoSpin, optimization conditions with PEG8000 at several concentrations (5%, 10%, and 25%) were evaluated for the morphology of cells and the binding aspect of the antibody–microparticles complex with target cells. At 25% PEG8000, cell morphology, especially granulocytes, was markedly altered. Overall, 10% PEG8000 was identified as the optimal concentration for ImmunoSpin. Images are shown at 400x magnification.

| 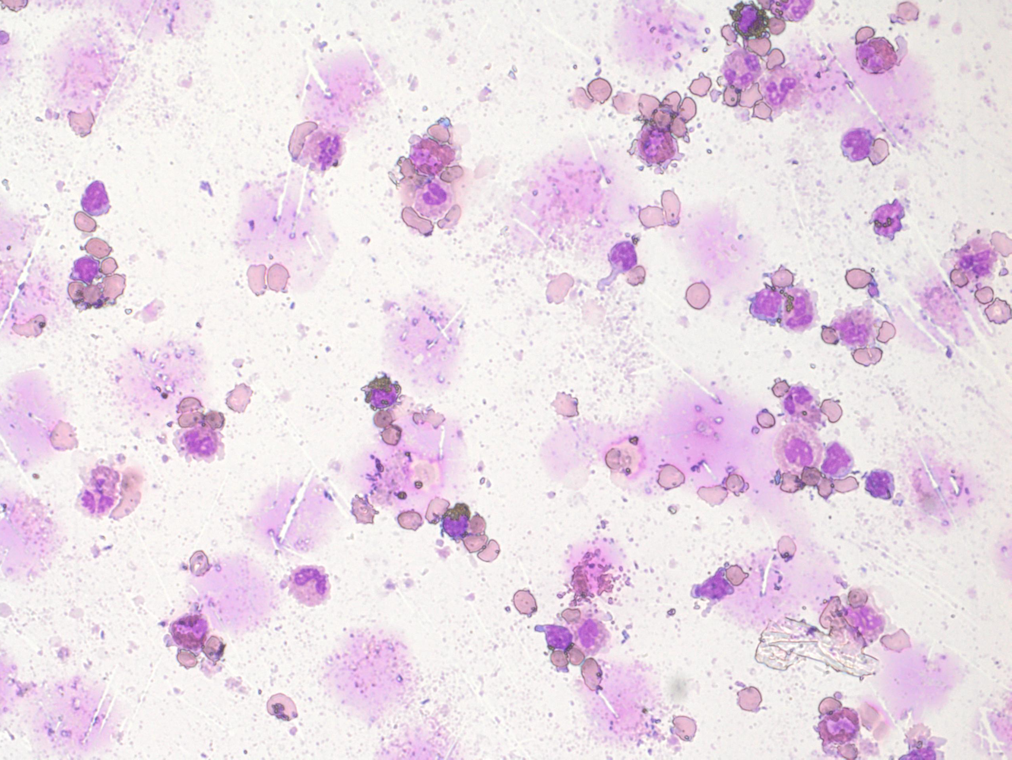 | 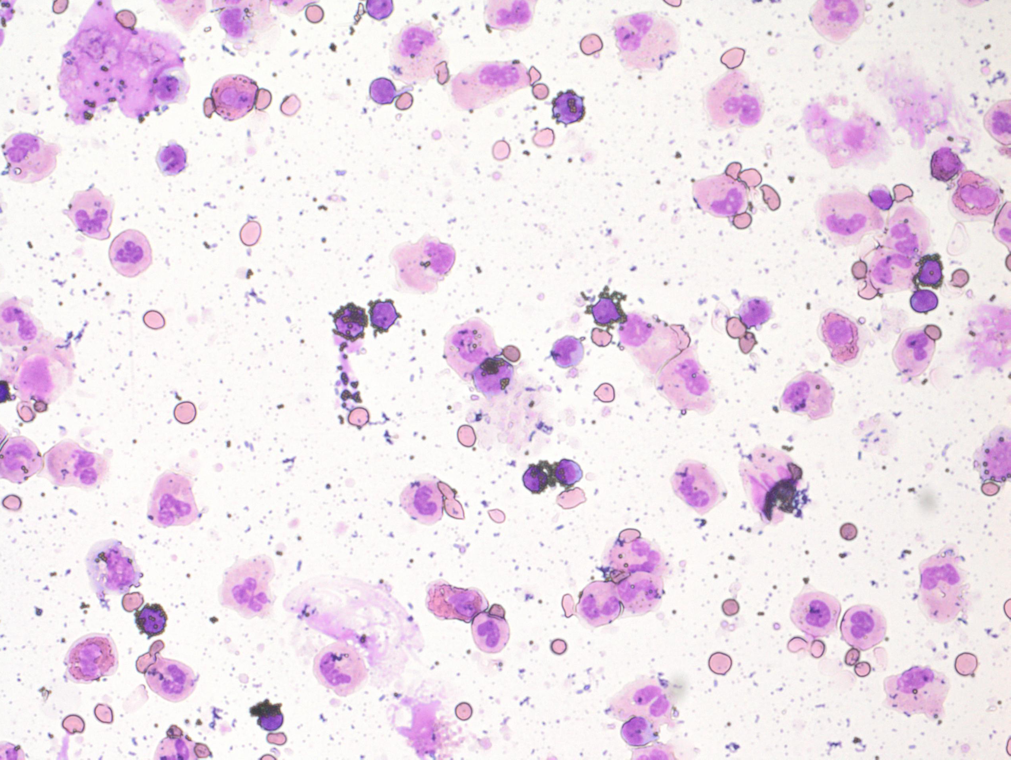 |
| --- | --- |
| No PEG8000, 24 h after cell suspension (400x) | 10% PEG8000, 24 h after cell suspension (400x) |

Supplementary Figure S4. Effect of 10% PEG8000 on ImmunoSpin treatment, which significantly decreased cell degradation at 24 h. After RBC lysis and supernatant removal, cell pellets were suspended in 10% PEG8000 PBS solution (800 µL) for cytospin. Cell suspension stability was evaluated 24 h after cell suspension preparation for cytocentrifugation with or without 10% PEG8000. Images are shown at 400x magnification.

| 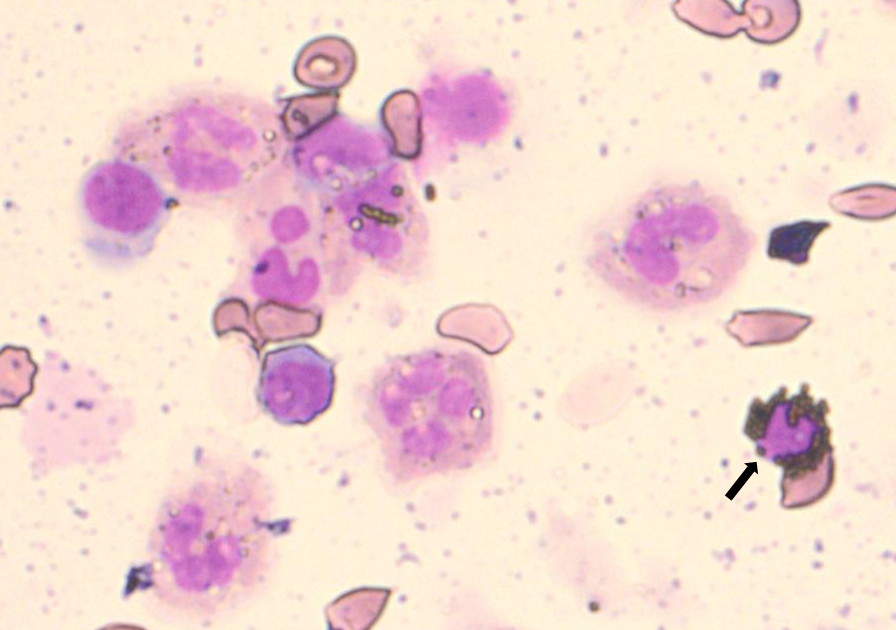 | 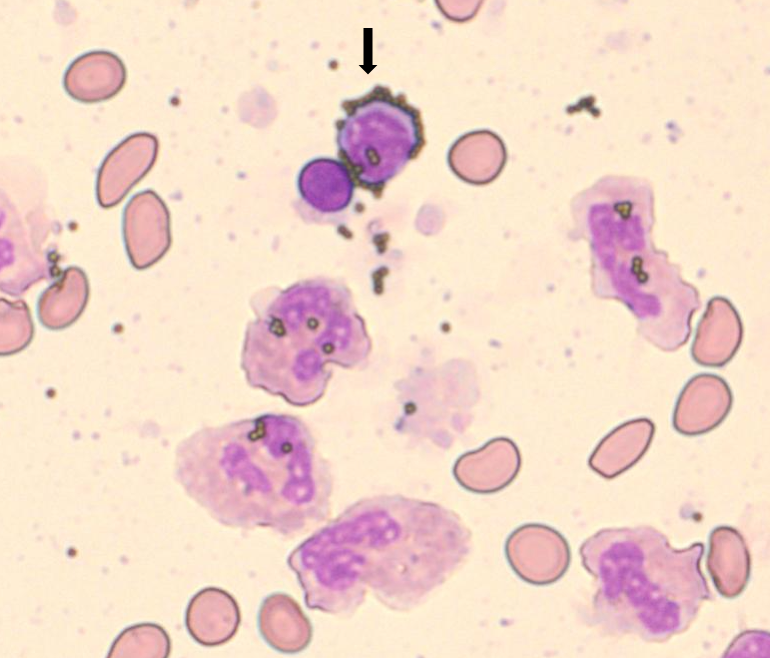 |
| --- | --- |

Supplementary Figure S5. No washing after RBC lysis. ImmunoSpin was performed without the RBC wash step. Microparticle-tagged CD4+ T cells were well-recognized (arrow) under a light microscope (400x, Wright stain). After RBC lysis, the washing step was not essential for ImmunoSpin.

**
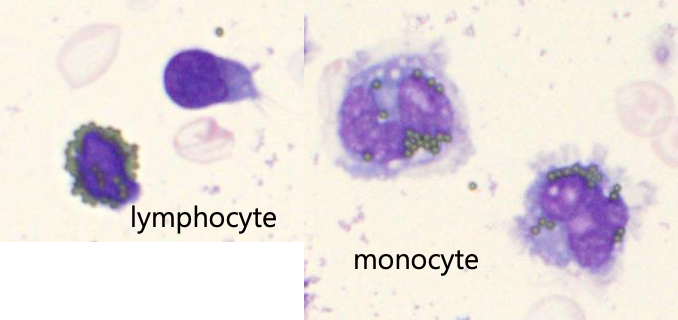
**

Supplementary Figure S6. Clear differential classification of CD4+ T lymphocytes from monocytes that bind the anti-CD4 antibody–microparticle complex (Wright stain). For CD4+ T cells, microparticles tagged along the cell membrane surface (left). However, microparticles were observed in the cytoplasmic area of the monocytes (right).

| 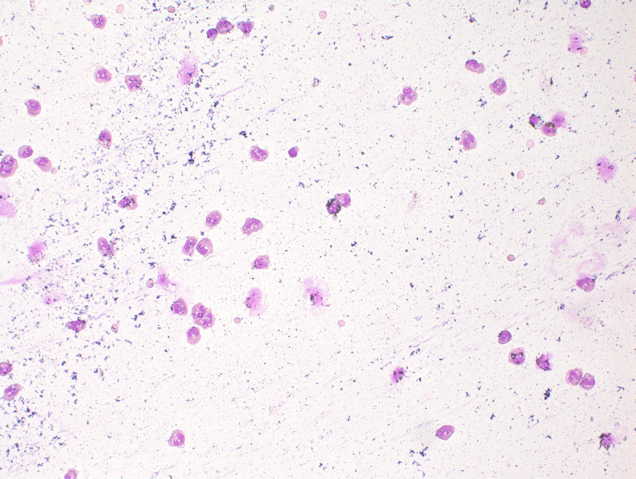 | 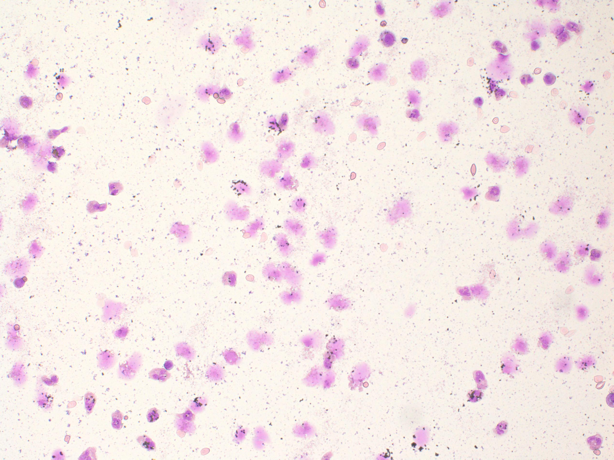 | 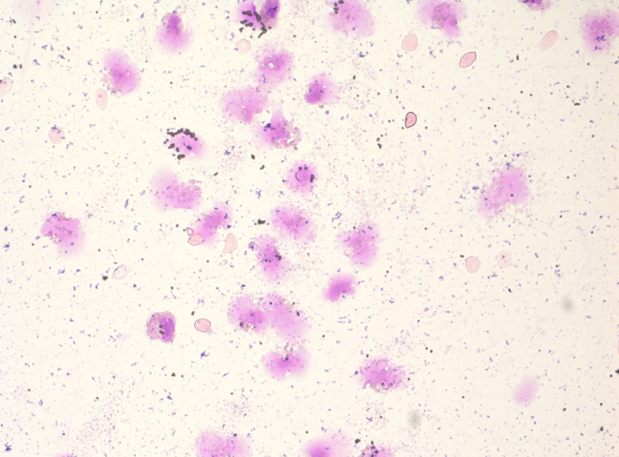 |
| --- | --- | --- |
| 10% Ficoll70, 0 h, (200x) | 10% Ficoll70, 3 h, (200x) | 10% Ficoll70, 3 h, (400x) |
| 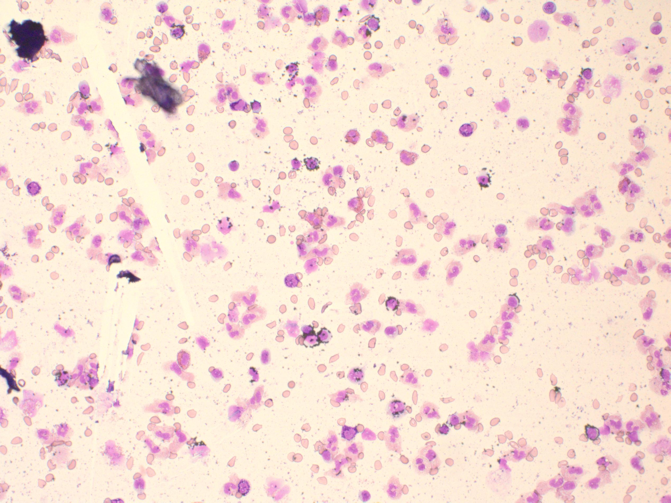 | 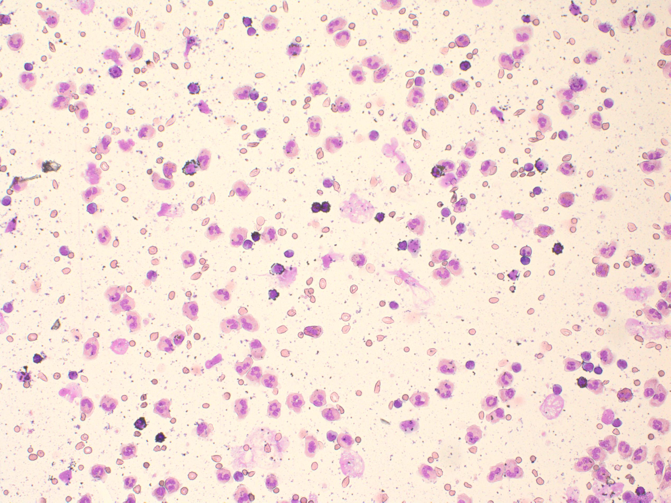 | 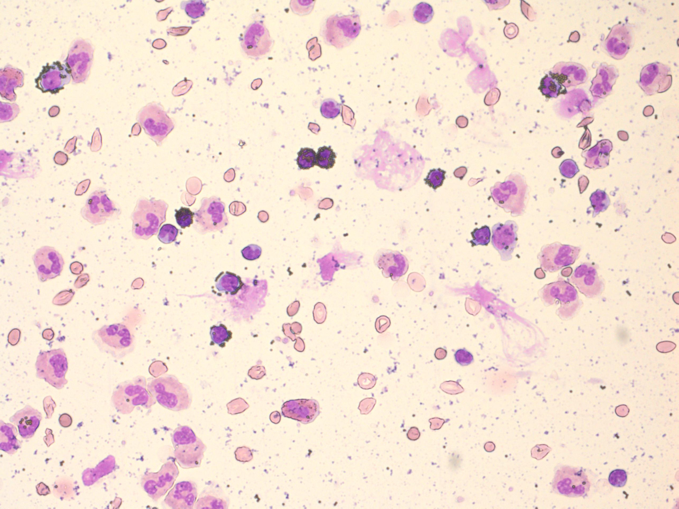 |
| 10% PEG8000, 0 h, (200x) | 10% PEG8000, 3 h, (200x) | 10% PEG8000, 3 h, (400x) |

Supplementary Figure S7. Effect of Ficoll70 on cell morphology. Before the cytospin step of ImmunoSpin, 10% Ficoll 70 kDa (Ficoll70, Sigma-Aldrich, St. Louis, MO, USA) was used to evaluate the morphology of cells and the binding aspect of the antibody–microparticles complex with target cells. There was no effect on the cell morphology and stability in comparison with 10% PEG8000. Images are shown with 200x and 400x magnifications.

|  | PBS + 10% PEG8000 | FACS buffer + 10% PEG8000 |
| --- | --- | --- |
| 0 hr | 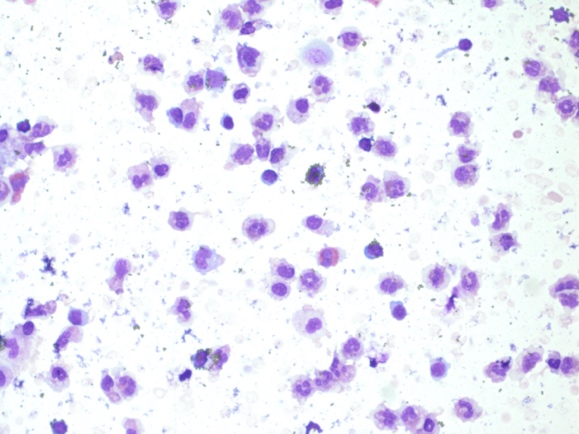 | 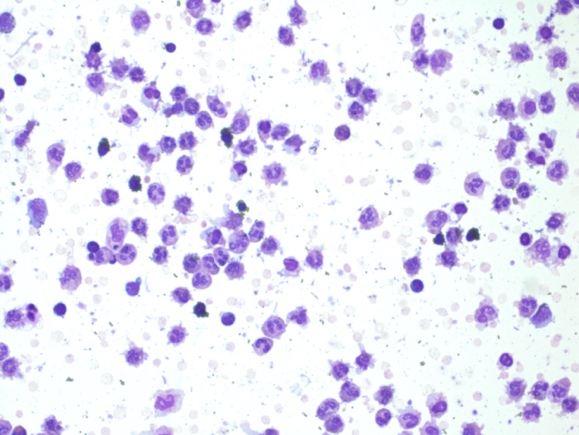 |
| 24 hr | 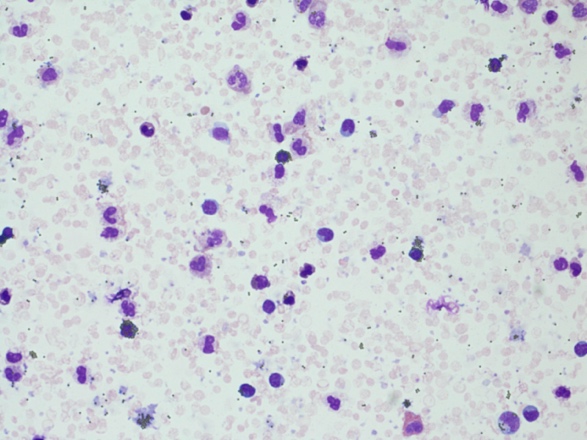 | 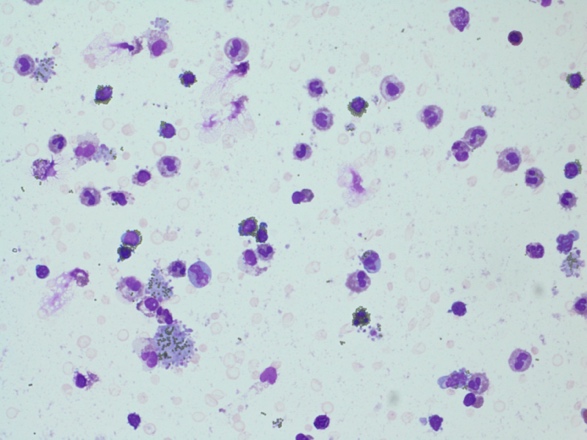 |

Supplementary Figure S8. Microscopic images (200x) of cytospin slides in cell suspension (0 h) and cell suspension stored for 24 h at room temperature either in 10% PEG8000 FACS buffer or 10% PEG8000 PBS buffer. Cell morphology was better preserved in 10% PEG8000 FACS buffer.

|  | FACS buffer with 10% PEG8000 | FACS buffer only | PBS with 10% PEG8000 |
| --- | --- | --- | --- |
| 0 hr | 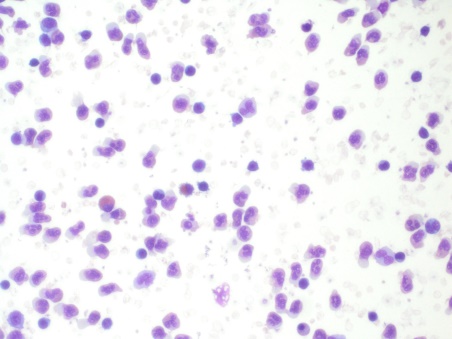 | 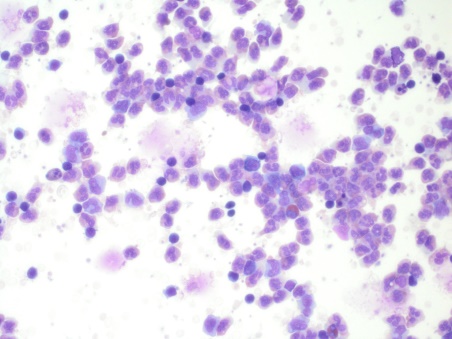 | 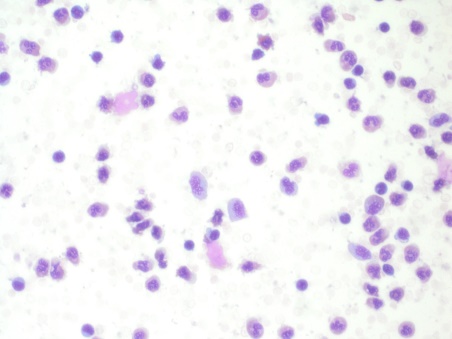 |
| 12 hr | 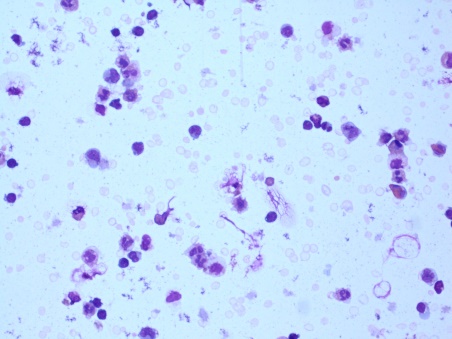 | 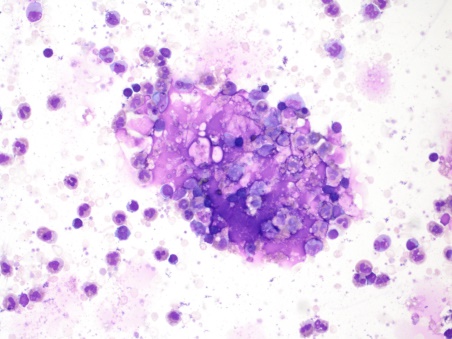 | 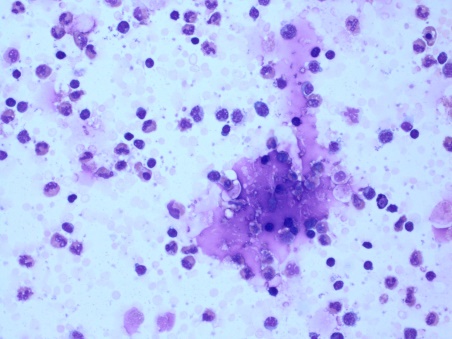 |
| 36 hr | 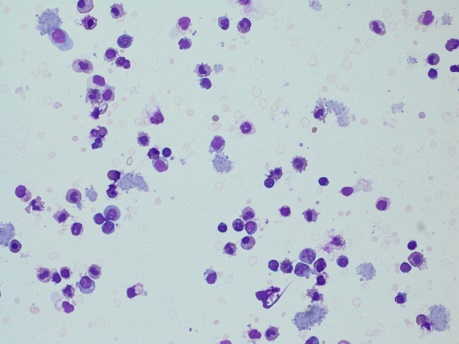 | 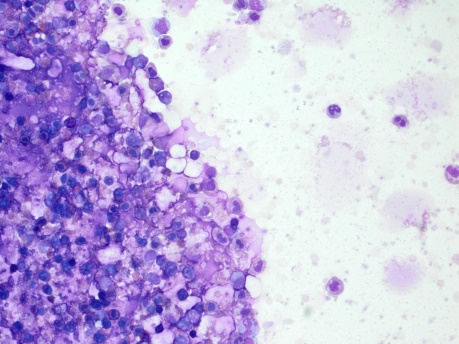 | 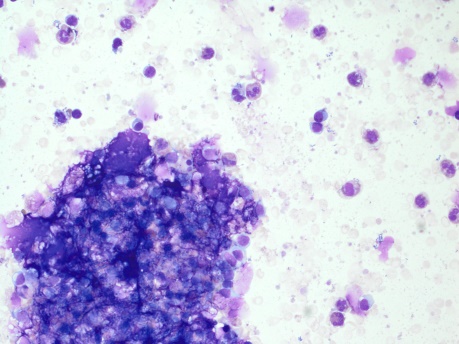 |

Supplementary Figure S9. Effect of FACS buffer and 10% PEG8000 on the preservation of cell morphology. After RBC lysis and supernatant removal, cell pellets were suspended in 10% PEG-8000 PBS, 10% PEG8000 FACS buffer, and FACS buffer only. Cytospin slides were prepared at 0, 12, and 36 h from the cell suspension. No anti-CD4 antibody–microparticle complex was added. Cell morphology was preserved in 10% PEG8000 FACS buffer.

| 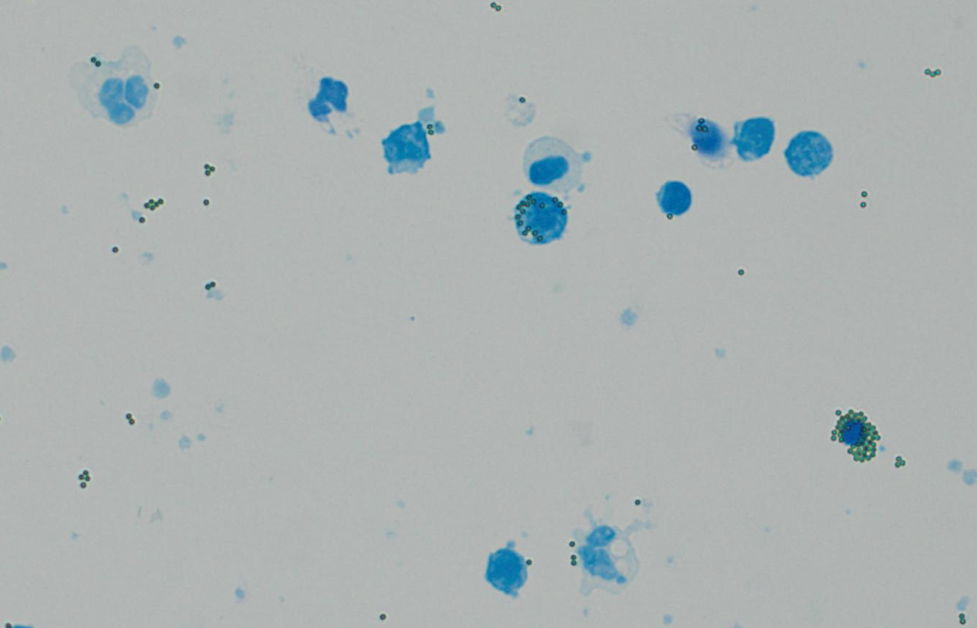 | 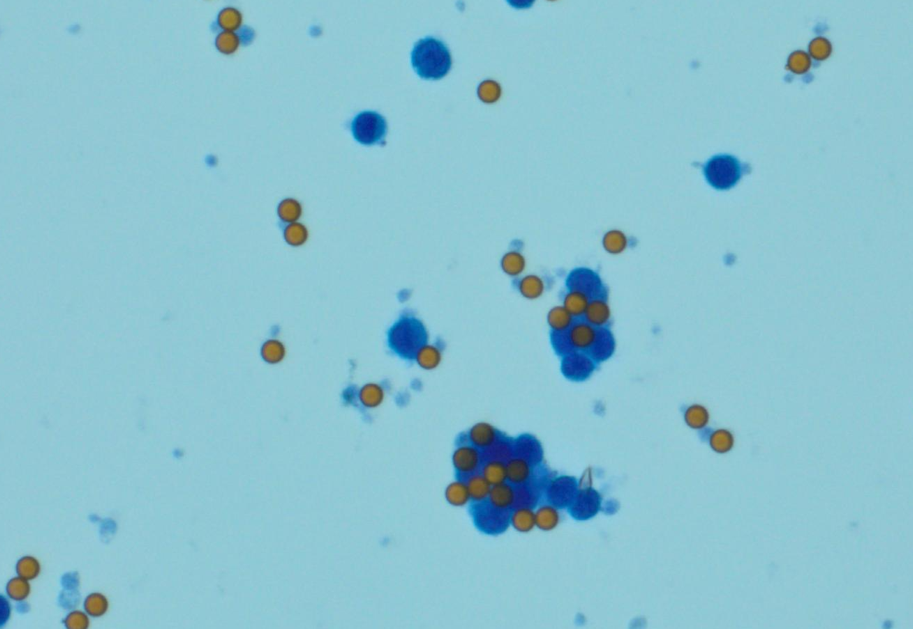 |
| --- | --- |
| 1 µm-sized microparticles (400x) | 4.5 µm-sized microparticles (400x) |

Supplementary Figure S10. Size effect of microparticles. Compared to 1 µm-sized microparticles (left), a larger size (4.5 µm diameter) of microparticle produced aggregation with target cells (right). Images are shown at 400x magnification (methylene blue staining).

References

1. Pant H, Beroukas D, Kette FE, Smith WB, Wormald PJ, et al. (2009) Nasal polyp cell populations and fungal-specific peripheral blood lymphocyte proliferation in allergic fungal sinusitis. Am J Rhinol Allergy 23:453-60.

2. Hultin LE, Chow M, Jamieson BD, O'Gorman MR, Menendez FA, et al. (2010) Comparison of interlaboratory variation in absolute T-cell counts by single-platform and optimized dual-platform methods. Cytometry B Clin Cytom 78:194-200.

3. Sarikonda G, Mathieu M, Natalia M, Pahuja A, Xue Q, et al. (2021) Best practices for the development, analytical validation and clinical implementation of flow cytometric methods for chimeric antigen receptor T cell analyses. Cytometry B Clin Cytom 100:79-91.

4. Kanakasabapathy MK, Pandya HJ, Draz MS, Chug MK, Sadasivam M, et al. (2017) Rapid, label-free CD4 testing using a smartphone compatible device. Lab Chip 17:2910-9.

5. Zeh C, Rose CE, Inzaule S, Desai MA, Otieno F, et al. (2017) Laboratory-based performance evaluation of PIMA CD4+ T-lymphocyte count point-of-care by lay-counselors in Kenya. J Immunol Methods 448:44-50.

6. Pham MD, Agius PA, Romero L, McGlynn P, Anderson D, et al. (2016) Performance of point-of-care CD4 testing technologies in resource-constrained settings: a systematic review and meta-analysis. BMC Infect Dis 16:592.

7. Larson B, Schnippel K, Ndibongo B, Long L, Fox MP, et al. (2012) How to estimate the cost of point-of-care CD4 testing in program settings: an example using the Alere Pima Analyzer in South Africa. PLoS One 7:e35444.

8. Wade D, Daneau G, Aboud S, Vercauteren GH, Urassa WS, et al. (2014) WHO multicenter evaluation of FACSCount CD4 and Pima CD4 T-cell count systems: instrument performance and misclassification of HIV-infected patients. J Acquir Immune Defic Syndr 66:e98-107.

9. Wade D, Diaw PA, Daneau G, Diallo AA, Mboup S, et al. (2015) Laboratory and field evaluation of the Partec CyFlow miniPOC for absolute and relative CD4 T-cell enumeration. PLoS One 10:e0116663.

10. Shafiee H, Wang S, Inci F, Toy M, Henrich TJ, et al. (2015) Emerging technologies for point-of-care management of HIV infection. Annu Rev Med 66:387-405.
